# The level of stress and body self image perception in adolescents with idiopathic scoliosis

**DOI:** 10.1186/1748-7161-9-S1-O62

**Published:** 2014-12-04

**Authors:** Edyta Kinel, Anna Podolska-Piechocka, Tomasz Kotwicki, Przemyslaw Lisinski

**Affiliations:** 1 Department of Rheumatology and Rehabilitation Clinic of Rehabilitation, University of Medical Sciences, Poznan, Poland; 2 Department of Paediatric Orthopaedics and Traumatology, University of Medical Sciences, Poznan, Poland

## Background

The level of stress and body self image perception in girls suffering from adolescent idiopathic scoliosis (AIS) is the object of interest of the professionals.

## Aim

Evaluation of the stress level in adolescents with AIS and their self image perception.

## Design

Observational study. It involved 82 adolescents, ages ranging between 11.0 and 16.0 years, all with IS with Cobb angle between 20-45 degrees. Adolescents were wearing the Chêneau orthosis, (more than 3 months, at least 12h per day). The age of examined group was 13.5 ± 1.6 years.

## Methods

The following were used in the evaluation: Bad Sobernheim Stress Questionnaire (Brace and Deformity), Trunk Appearance Perception Scale (TAPS), and Knee – Feet Perception Scale (KFPS). The BSSQ Brace estimates the stress scoliosis patients have whilst wearing brace, the BSSQ Deformity estimates the stress involved with body deformation. Minimal points number equals 0 (the greatest stress), maximal 24 (the least stress). The TAPS evaluates subjective impression of trunk deformity from 3 viewpoints: looking toward the front, back, and with the patient bending over. Minimal points number equals 1 (the biggest deformity), maximal equals 5 (the least deformity). KFPS estimates subjective impression of knee joints and feet position. Minimal points number equals 1 (the biggest deformity), maximal equals 3 (the least deformity). Additionally results of TAPS and KFPS were compared with clinical examination.

## Results

Cobb angle was 31.0 ± 8.1 degrees. The BSSQ Brace and Deformity median was 13, the TAPS median of the total score was 4, the KFPS median of the total score was 3. There were significant differences between Cobb angle and TAPS (AIS) and Cobb angle and TAPS clinical examination (p<0.001).

## Conclusions

Conservative treatment does not severely impact on the level of stress and the self image of adolescents with idiopathic scoliosis.
